# Chronic Consumption of Sweeteners and Its Effect on Glycaemia, Cytokines, Hormones, and Lymphocytes of GALT in CD1 Mice

**DOI:** 10.1155/2018/1345282

**Published:** 2018-04-24

**Authors:** Cristian Angel Rosales-Gómez, Beatriz Elina Martínez-Carrillo, Aldo Arturo Reséndiz-Albor, Ninfa Ramírez-Durán, Roxana Valdés-Ramos, Talia Mondragón-Velásquez, Jorge Alberto Escoto-Herrera

**Affiliations:** ^1^Laboratorio de Investigación en Nutrición, Facultad de Medicina, Universidad Autónoma del Estado de México, Paseo Tollocan, Esquina Jesús Carranza s/n, Colonia Moderna de la Cruz, 50180 Toluca, MEX, Mexico; ^2^Laboratorio de Inmunología de Mucosas, Escuela Superior de Medicina, Instituto Politécnico Nacional, Plan de San Luis y Díaz Mirón, 11340 Ciudad de México, Mexico; ^3^Laboratorio de Microbiología Medicina y Ambiental, Facultad de Medicina, Universidad Autónoma del Estado de México, Paseo Tollocan, Esquina Jesús Carranza s/n, Colonia Moderna de la Cruz, 50180 Toluca, MEX, Mexico

## Abstract

**Background:**

The consumption of sweeteners has increased in recent years, being used to control body weight and blood glucose. However, they can cause increased appetite, modification of immune function, and secretion of hormones in the GALT.

**Objective:**

To assess the effect of chronic sweetener consumption on glycaemia, cytokines, hormones, and GALT lymphocytes in CD1 mice.

**Material and Methods:**

72 CD1 mice divided into 3 groups were used: (a) baseline, (b) middle, and (c) final. Groups (b) and (c) were divided into 4 subgroups: (i) Control, (ii) Sucrose, (iii) Sucralose, and (iv) Stevia. The following were determined: body weight, hormones (GIP, insulin, and leptin), lymphocytes CD3^+^T cells and CD19^+^B cells, IgA^+^ plasma cells, and cytokines (IL-4, IL-5, IFN-*γ*, and TNF-*α*).

**Results:**

Sucralose reduces secretion of GIP and glycaemia but does not modify insulin concentration, increases body weight, and reduces food intake. Stevia increases the secretion of GIP, insulin, leptin, body weight, and glycaemia but keeps food consumption normal. Sucralose and Stevia showed a higher percentage of CD3^+^T cells, CD19^+^B cells, and IgA^+^ plasma cells in Peyer's patches, but only Stevia in lamina propria.

**Conclusion:**

Sweeteners modulate the hormonal response of cytokines and the proliferation of lymphocytes in the intestinal mucosa.

## 1. Introduction

The increase in the rate of overweight and obesity worldwide has generated the need to seek new treatment and prevention strategies [1]. One of them has been the widespread use of sweeteners in the population in order to reduce caloric intake, body weight, and blood glucose levels and thus prevent the development of chronic noncommunicable diseases [2, 3]. Sweeteners are additives that provide sweetness to food and drinks and mimic the sweet effect of sugar [2]. Sweeteners can be classified by their origin in natural (sucrose and stevia) and/or artificial (sucralose), and also as nutritive for having an energy intake similar to sugar and nonnutritive for not providing energy to the body [4]. The use of sweeteners is not toxic to health. However, it has been observed that they exert diverse effects on some cellular pathways [5]. For example, sucralose in studies* in vitro* inhibits the inflammatory response, causing a decrease in the humoral response which can cause an increase in susceptibility against external pathogens [6]. Moreover, nutritive sweeteners such as sucrose enhance the cellular inflammatory response that may favor the defense against infectious agents [7].

Now, the intestinal mucosa is an interface between the inside and outside of the organism; it is in direct contact with a large number of molecules or agents foreign to the organism contained in food, for which it has a great capacity to discern between harmful agents and innocuous [8]. Mucosal immunity maintains selective absorption and intestinal barrier function despite continuous antigenic stimulation, discriminating between pathogens and harmless antigens of the diet [9]. An important element in this process is the Gut Associated Lymphoid Tissue (GALT). It is the most important induction site of the mucosal immune system, formed by organized and specialized lymphoid tissue. It contains well-defined organs such as Peyer's patches (inductor compartment), oval and irregular, that are located along the distal ileum [10] and separated from the intestinal lumen by M epithelial cells or enterocytes specialized in the uptake of luminal antigens [11].

In the GALT, there are also cellular aggregates that comprise the diffuse lymphoid tissue: mesenteric lymph nodes and scattered lymphoid cells that are distributed in two compartments, epithelium and intestinal lamina propria (effector compartment): in the lamina propria scattered: macrophages, dendritic cells, plasma cells, T helper lymphocytes, and, in a lesser proportion, eosinophils [12]. The main cellular population is located in the small intestine, corresponding to intraepithelial lymphocytes (T cytotoxic) [13] and plasma cells that produce IgA [11, 14].

Among the natural sweeteners, the most used in the market is the stevia. It has been reported that steviol glycosides increase the activity of phagocytes, the haemagglutination of antibodies, and delayed hypersensitivity, considerably increasing the proliferation of B and T cells stimulated by lipopolysaccharide (LPS). Therefore, they are considered immunomodulatory agents activating humoral immunity, cellular immunity, and phagocytic function [15]. On the other hand, supplementation with steviosides at a dose of 300 mg/kg of body weight reduces the secretion of proinflammatory interleukins IL-1*β*, IL-6, and TNF-*α* and inhibits the expression of TLR2 receptors [16]. Other studies suggest that stevia extract* (Rebaudioside A)* increases insulin secretion not by the action of incretin hormones but, rather, by the inhibition of ATP-dependent potassium channels and suppressing the secretion of Glucagon by alpha cells of the pancreas [17–19].

Although nonnutritive sweeteners have been considered metabolically inert, recent data suggest that these may have physiological effects that alter glucose metabolism and stimulate appetite [20]. Much of this research is based on the discovery of sweet taste receptors T1r2 and T1r3 in oropharynx and enteroendocrine cells of the intestine and pancreas. These, in turn, stimulate the secretion of Glucose Insulinotropic dependent Peptide (GIP) [21, 22].

In patients with type 2 diabetes mellitus, it has been reported that the use of sucralose does not modify glycosylated hemoglobin concentrations, but it may stimulate the intestinal absorption of glucose by stimulating flavor receptors and GLUT2 receptors [23]. Ford et al. [24] reported that oral administration of sucralose in healthy patients does not affect the secretion of GIP. In a study where sucrose and sucralose were administered to rats, an increase in GIP was observed only in the group supplemented with sucrose [25]. In patients with obesity diagnosis that were supplemented with sucralose through a nasogastric tube, GIP concentrations did not increase [22]. Therefore, the hypothesis of this work is whether the chronic consumption of sweeteners (natural and artificial) affects glycaemia, the concentration of cytokines (anti- and proinflammatory), the secretion of hormones such as GIP and insulin, and the percentage of lymphocytes in GALT of CD1 mice.

## 2. Material and Methods

### 2.1. Study Design

The present experimental, prospective, controlled, and randomized study was conducted with 21-day-old CD1 mice obtained from the bioterium of the Facultad de Medicina, Universidad Autónoma del Estado de México (UAEM). Animal care and experimental procedures were carried out in accordance with the standards of the Internal Regulation for the Use of Lab Animals and the Ethical Investigation Committee of the UAEM, as well as the guidelines of the Mexican Secretary of Health for the Production and Care of Lab Animals (NOM-062-ZOO-1999 Ministry of Agriculture, Mexico City, Mexico). Animals were hosed in individual cages during the entire experiment and food was offered ad libitum (from the 4th to the 12th week of life). All animals were maintained on a 12/12 h light/dark cycle, at 21°C of temperature.

The animals were fed with a standard normal diet (Rodent Laboratory Chow 5001 of Purina [3.02 Kcal/g]). The study was carried out in the Nutrition Research Laboratory and Mucosal Immunology Laboratory, School of Medicine in the Instituto Politécnico Nacional.

### 2.2. Study Groups

We used 72 mice, divided into 3 groups according to the time of supplementation: (a) baseline group (*n* = 8), three weeks old, freshly weaned without treatment, (b) middle (*n* = 32), nine weeks old, with six weeks of treatment, and (c) final (*n* = 32), 15 weeks old, with 12 weeks of treatment. Groups (b) and (c) were divided into 4 subgroups according to the type of sweetener administered (*n* = 8): (i) Control (administration of water without sweetener), (ii) Sucrose, (iii) Sucralose, and (iv) Stevia. Table sugar (sucrose) and artificial (sucralose) and natural sweeteners (stevia) were used.

### 2.3. Preparation and Administration of Water with and without Sweetener

The sweetener was diluted in ultrapure water with the following concentrations: sucrose 41.66 mg/mL, sucralose 4.16 mg/mL, and stevia 4.16 mg/mL. The control subgroup without sweetener was given only ultrapure water throughout the treatment. The solutions with sweetener were placed in the waterers in a schedule of 8–13 h (5 h). Subsequently, the drinkers were removed and replaced with water without sweetener until the following day. The volume of water with and without sweetener was quantified daily to determine the consumption preference.

### 2.4. Determination of Body Weight, Food Consumption, and Glycaemia

The body weight, food consumption, and glycaemia of mice were quantified weekly from the 3rd week until the 15th week of age. For the determination of glycaemia, the mouse was anesthetized with ether vapors. A One Touch glucometer (Bayer) was used; the sample was taken by capillary puncture in the tail vein at 7:30 a.m., prior to administration of the water with sweeteners.

### 2.5. Collection and Determination of Blood Samples

At the end of the 15th week of life, the animals were anaesthetized with pentobarbital (80 mg/kg), bled by direct cardiac puncture (using a syringe with heparin), and sacrificed by cervical dislocation. Whole blood was used, which was centrifuged for 10 minutes at 2500 rpm to separate the two blood phases. Serum was collected and transferred to Eppendorf tubes to quantify GIP, insulin, and leptin. The determinations were performed through Luminometry with a Luminex 201 of Millipore™, with a commercial Kit (Mouse Metabolic Magnetic Bead Panel, No. Cat. MMHMAG-44K) of Milliplex® Map, following the provider recommendation.

### 2.6. HOMA Index

To determine the sensitivity and degree of insulin resistance, the HOMA index was determined by applying the following formula [26]:

(1) HOMA=Glycaemia  mg/dL/18.2×InsulinmU/mL22.517.

### 2.7. Isolation of Lymphocytes from Small Intestine

#### 2.7.1. Lamina Propria

The isolation of lymphocytes from Peyer's patches and lamina propria from the small intestine was carried out as previously described [27] with brief modifications. Small intestine (SI) segment was dissected, carefully cleaned from its mesentery, and flushed of fecal contents with 5 mL of PBS-1X (Phosphate Buffered Saline 1X). Peyer's patches were carefully removed from the small intestine before processing. Then the SI were everted by introducing a 10 cm long iron crochet needle tied to a string. The intestine was tied up at one end, the crochet needle removed, and the string pulled carefully while the intestine was kept immersed in cold RPMI-1640 medium (Sigma-Aldrich, USA, Cat. R6504). Each everted intestinal segment was transferred to a 50 ml tube containing 25 ml of RPMI medium with 60 U/ml of type IV collagenase (Sigma-Aldrich, USA, Cat No. C5138), DTT (1,4 Dithiothreitol, Sigma-Aldrich, USA, Cat No. 43819), 1% FCS and 50 *μ*g/ml gentamicin. The tubes were incubated horizontally for 30 min at 37°C in a shaking-water bath at 150 rpm. The contents of each tube were then transferred to Petri dishes and 200 *μ*l FCS was added. The intestinal mucosa was compressed with a syringe plunger over a plastic mesh; single cell suspensions containing lamina propria cells were filtered through organdy mesh and then centrifuged for 10 min at 1500 rpm at 4°C. Cell suspensions were collected and centrifuged in a discontinuous 40%/70% Percoll gradient at 2500 rpm for 25 min. Cells from the interface were washed and suspended in RPMI medium.

#### 2.7.2. Peyer's Patches

After the small intestine was separated, Peyer's patches were sheared from the small intestine, triturated in 3% fetal calf serum (FCS)/PBS solution on ice, and filtrated via a 300 section stainless steel cell strainer to obtain lymphocytes. Cells were centrifuged at 1500 rpm at 4°C for 10 min.

#### 2.7.3. Flow Cytometry Assays

Cells suspensions of Peyer's patches and lamina propria were adjusted to 1 × 10^6^ cells/mL in PBS for cytofluorometric analysis with brief modifications [28]. (i) Surface phenotype of T cells was detected by using fluorescent labeled monoclonal antibodies: anti-CD3 FITC (Cat. No. 553063), anti-CD45/B220 (PerCP, Cat. No. 553093), and anti-CD19 (PE, Cat. No. 553786) (all from BD Biosciences). Cells were incubated for 30 min at room temperature. Finally, the cells were then washed with PBS and fixed in 1% paraformaldehyde. (ii) The percentage of IgA^+^ plasma cells was detected by the addition of a cocktail containing anti-CD19 PE, anti-CD138 APC, and anti-IgA FITC antibodies (all from BD Biosciences, San Jose, CA, USA). Plasma cells and B cells were fixed, permeabilized, and stained according to BD Bioscience's protocol for intracellular staining. (iii) For the detection of intracellular cytokine production, lymphocytes were stimulated with a mixture containing phorbol myristate acetate, ionomycin, and Brefeldin A (Leucocyte Activation Cocktail Kit, BD Pharmingen) and incubated for 4 h at 37°C and 5% CO_2_. Then, antibodies to cell surface markers, anti-CD4 PerCP, were added and incubated as before. For intracellular staining of CD4^+^ T cells, fixation and permeabilization were performed using Cytofix/Cytoperm Kits (BD Pharmingen) according to the manufacturer's instructions. These cells were incubated with anti-IL-4 PE (Cat. No. 554435), anti-IL-5 PE (Cat. No. 554395), anti-IL-10 FITC (Cat. No. 554466), anti-IFN-*γ* FITC (Cat. No. 554411), and anti-TNF-*α* PE antibodies (Cat. No. 554419). The fluorescent signal intensity was recorded and analyzed by FACS Aria Flow Cytometer (Becton Dickinson). Events were collected from the lymphocyte gate on the FSC/SSC dot plot. 20,000 gated events were acquired from each sample using the CellQuest research software (Becton Dickinson). Data was analyzed using Summit software v4.3 (Dako, Colorado Inc.). Data from eight mice per group are reported as the mean ± standard deviation (SD).

### 2.8. Statistical Analysis

Based on the homogeneity of the data, the mean ± SD and one-way ANOVA variance analysis were used, with Tukey's HSD post hoc test to perform comparisons by type of treatment (control, sucrose, sucralose, and stevia) between the subgroups and repeated measures ANOVA to compare the groups by treatment time (3, 9, and 15 weeks of treatment). The Friedman test and the Kruskal-Wallis H test were used to compare the median values that demonstrated a nonnormal distribution. The differences were considered significant with a value of *p* < 0.05. Data were analyzed using SPSS 19 statistical software for Windows.

## 3. Results

### 3.1. Consumption of Water with and without Sweeteners

#### 3.1.1. Consumption of Water with Sweeteners

The consumption of water with sweetener at the 4th week is higher in the subgroups of Sucralose and Stevia (*F* = 268.98, *p* = 0.001). The same behavior was observed at the 9th (*F* = 502, *p* = 0.001) and 15th week (*F* = 4816, *p* = 0.001). The consumption preference is shown with sucralose, followed by stevia (Table 1). When comparing the groups by treatment time, the differences were significant (repeated measures ANOVA *F* = 7184, *p* = 0.001), with a higher preference for water consumption with sucralose and stevia.

#### 3.1.2. Mice Show Preference for Water Consumption with Sucralose and Stevia

The consumption of water without sweetener at week 4 shows significant differences (*F* = 7.58, *p* = 0.001). Water intake was increased in the Stevia subgroup compared to Sucrose (HSD Tukey, *p* = 0.006) and Sucralose (HSD Tukey, *p* = 0.001). By the 9th week, water consumption remained unchanged. However, at week 15, differences (*F* = 3.11, *p* = 0.042) were observed in the higher water intake in the Stevia subgroup (HSD Tukey, *p* = 0.042) compared to the Sucrose group (Table 1). When comparing the groups, the difference was significant (repeated measures ANOVA, *F* = 1058, *p* = 0.001) at the 4th, 9th, and 15th week, between the Sucrose and Stevia groups (HSD Tukey, *p* = 0.003). Animals of Sucralose and Stevia subgroups consume more volume of water with sweetener during the 12 weeks.

### 3.2. Determination of Body Weight, Food Consumption, and Glycaemia

The baseline body weight of the mice at week 3 was 9.27 g. At the 9th and 15th week of supplementation, body weight did not show statistically significant differences in any of the study groups as shown in Table 2. The differences were found when comparing the body weights of the groups by time of supplementation at 3, 9, and 15 weeks of age (*F* = 1935, *p* = 0.001), where body weight increased with consumption of sucralose and stevia at the end of week 15th.

Regarding blood glucose, a progressive increase in concentration was found at the 9th and 15th week compared to the basal group (*F* = 13.04, *p* = 0.001). The increase was proportional to the time of consumption of the sweetener. No differences were observed in the glycaemia of the subgroups at 9th (one-way ANOVA, *F* = 0.782, *p* = 0.514) and 15th (one-way ANOVA *F* = 0.940, *p* = 0.435) weeks of age (Table 2).

The consumption of food at the 4th week was not modified (Table 2). At 9 week of age, feed intake is significantly reduced in the Stevia subgroup (HSD Tukey *p* = 0.042). However, in week 15, the reduction is observed in the subgroup of Sucrose (HSD Tukey *p* = 0.009) and Stevia (HSD Tukey *p* = 0.017) compared with the control subgroup, as shown in Table 2. Differences were found when comparing the groups by the time they consumed the sweetener (*F* = 505.46, *p* = 0.001). The animals of the 15-week-old group who consumed the sweetener for 12 weeks reduced their food intake (Table 2).

### 3.3. Hormone Profile in CD1 Mice That Consumed Sweeteners for 12 Weeks

Gastric Inhibitor Peptide (GIP) was quantified; no differences were found between the subgroups at weeks 9 and 15 of age. The comparison of the groups by time of supplementation was significant (*p* = 0.001); the stevia group increased the secretion of GIP at the 9th and 15th weeks (Table 3).

Insulin secretion at the 9th week is increased in the subgroups of Sucralose and Stevia. This increase is notable at week 15 in the sucrose and stevia subgroups. When comparing the groups by treatment time, the differences are not significant (Friedman, *p* = 0.055) as shown in Table 3.

The leptin concentration increases in the Stevia subgroup at the 9th week, but the differences are not significant (Kruskal-Wallis Test *p* = 0.397). This increase is observed in all subgroups at week 15. When the groups were compared by treatment time, the differences were not significant (Friedman, *p* = 0.055), as shown in Table 3.

The HOMA index was determined to assess the level of insulin resistance of mice. Consumption of stevia increases insulin resistance at 9 weeks and is significantly reduced with the consumption of sucrose (Kruskal-Wallis test *p* = 0.016). Stevia continues to increase this index in week 15, although there is no statistical significance (Kruskal-Wallis test *p* = 0.757). When comparing the groups by time of supplementation, significant differences were found; stevia increased the index progressively (Friedman, *p* = 0.008), as shown in Table 3.

### 3.4. Lymphocytes of the Lymphoid Tissue Associated with Intestine

#### 3.4.1. Lymphocytes Obtained from Peyer's Patches of the Small Intestine

**Figure 1:**
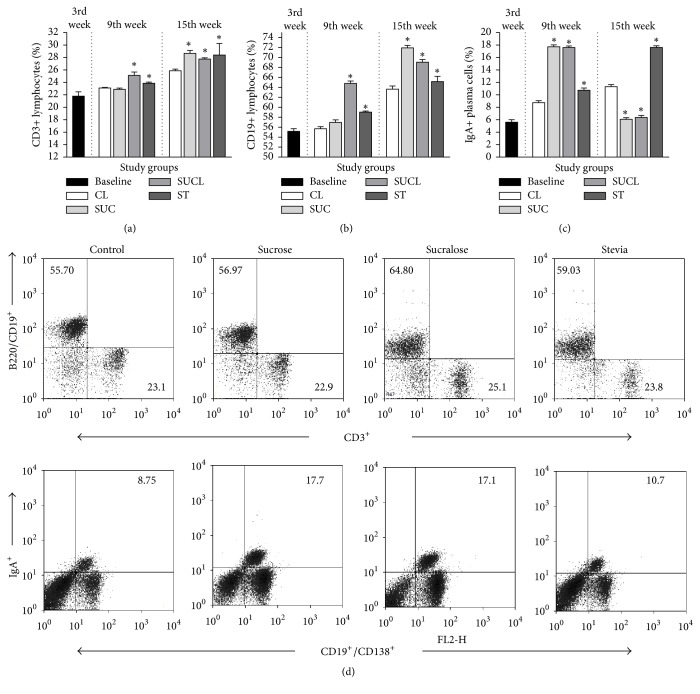
Percentage of Peyer's patches lymphocytes from the small intestine of CD1 mice, supplemented with sweeteners at 3 (baseline), 9 (middle), and 15 (final) weeks of age. (a) CD3^+^ lymphocytes, (b) CD19^+^ lymphocytes, and (c) IgA^+^ plasma cells. The values represent the mean ± SD. One-way ANOVA^*∗*^ was performed to compare the differences between the subgroups. The differences were considered significant with a value of *p* < 0.05^*∗*^. CL (Control), SUC (Sucrose), SUCL (Sucralose), and ST (Stevia). Peyer's patches B and T cells from the small intestine of CD1 mice supplemented with sweeteners for 9th weeks. (d) Representative Dot-Plots of CD3^+^, CD19^+^/B220^+^, and IgA^+^ plasma cells on lamina propria lymphocytes isolated from small intestine at 9th weeks of age supplemented with sweeteners as described in Material and Methods.

In Peyer's patches of the small intestine, the percentage of CD3^+^ T cells (9th: 25.15 ± 0.539; 15th: 27.76 ± 0.208) and CD19^+^ B cells (9th: 64.8 ± 0.491; 15th: 69.07 ± 0.539) increased (*p* = 0.001) with sucralose consumption, but decreased in the IgA^+^ plasma cells (9th: 17.16 ± 0.267; 15th: 6.37 ± 0.320) as shown in Figures 1(a), 1(b), and 1(c) respectively. On the other hand, stevia increased significantly CD3^+^ T cells (9th: 23.87 ± 0.192; 15th: 28.24 ± 1.85), CD19^+^ B cells (9th: 59.03 ± 0.229; 15th: 65.18 ± 1.03), and IgA^+^ plasma cells (9th: 10.74 ± 0.374; 15th: 17.63 ± 0.267) as shown in Figures 1(a), 1(b), 1(c), and 1(d).

#### 3.4.2. Lymphocytes Obtained from Lamina Propria of the Small Intestine

**Figure 2:**
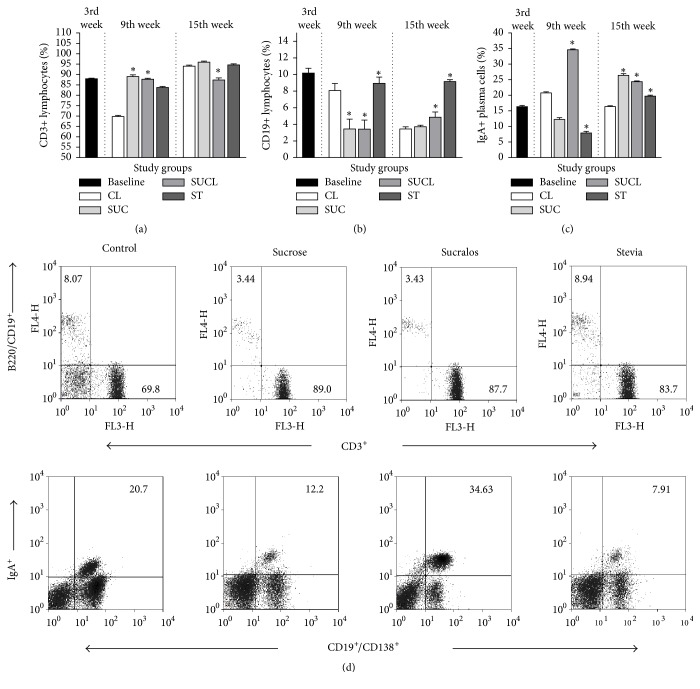
Percentage of lamina propria lymphocytes from the small intestine of CD1 mice, supplemented with sweeteners at 3 (baseline), 9 (middle), and 15 (final) weeks of age. (a) CD3^+^ lymphocytes, (b) CD19^+^ lymphocytes, and (c) IgA^+^ plasma cells. The values represent the mean ± SD. One-way ANOVA^*∗*^ was performed to compare the differences between the subgroups. The differences were considered significant with a value of *p* < 0.05^*∗*^. CL (Control), SUC (Sucrose), SUCL (Sucralose), and ST (Stevia). Lamina propria B and T cells from the small intestine of CD1 mice supplemented with sweeteners for 9th weeks. (d) Representative Dot-Plots of CD3^+^, CD19^+^/B220^+^, and IgA^+^ plasma cells on lamina propria lymphocytes isolated from small intestine at 9th weeks of age supplemented with sweeteners as described in Material and Methods.

*CD3* ^*+*^ *T Cells*. The percentage of CD3^+^ T cells increased in the 9th week of age in all groups (Tukey HSD, *p* = 0.001) compared with the control. At week 15, only sucralose decreased the percentage of CD3^+^ T cells (HSD Tukey, *p* = 0.003). When the groups were compared at 3, 9, and 15 weeks of age, an increase in the percentage of lymphocytes was observed (*F* = 173, *p* = 0.001), in the Sucrose and Stevia subgroups, without modification in the Sucralose subgroups (Figure 2(a)). 

*CD19* ^*+*^ *B Cells*. The CD19^+^ B cells decreased in the subgroups of Sucrose and Sucralose at 9th weeks of age (*p* = 0.001), but increased in week 15 in the subgroups of Sucralose and Stevia (*p* = 0.001). When comparing the groups by time of treatment, an increase in the percentage of CD19^+^ B cells was observed in the subgroups of Sucralose and Stevia (*F* = 348, *p* = 0.001) as shown in Figures 2(b) and 2(d). 

*IgA* ^*+*^ *Plasma Cells*. The subgroup of Sucralose increased the percentage of IgA^+^ plasma cells in lamina propria at the 9th week of age (HSD Tukey, *p* = 0.001). On the other hand, the subgroups of Sucrose and Stevia decreased their percentage of lymphocytes, compared with the control (*p* = 0.001). At week 15 of age, all subgroups increased the IgA^+^ plasma cells (*p* = 0.001) compared to the control. When comparing the 3rd, 9th, and 15th weeks of treatment groups, differences were observed (*F* = 203, *p* = 0.001) with an increase in the production of IgA^+^ plasma cells in all the groups (Figures 2(c) and 2(d)).

### 3.5. Cytokines

#### 3.5.1. Concentration of Intracellular Cytokines Determined in Peyer's Patches Lymphocytes

*Proinflammatory Cytokines (Th1)*. The percentage of IFN-*γ* decreased with the prolonged consumption of sweeteners (12 weeks) in the subgroups of Sucralose and Stevia (HSD Tukey, *p* = 0.001), as shown in Table 4. In contrast, the production of TNF-*α* increased with the consumption of sucralose and stevia (HSD Tukey *p* = 0.001) at the end of week 15 of age (Table 4). When the percentages of proinflammatory cytokine-producing IFN-*γ* and TNF-*α* were compared at the 3rd, 9th, and 15th weeks, it was observed that the percentage of these intracellular cytokines was reduced in the subgroups of Sucralose and Stevia compared with the baseline group (*F* = 1237, *p* = 0.001). (Table 4).

*Anti-Inflammatory Cytokines (Th2)*. The CD4^+^ T cells producing IL-4 at 9 weeks of age decreased in the Sucrose and Stevia subgroups, but increased with the intake of sucralose (HSD Tukey *p* = 0.001). The behavior changed after 12 weeks of treatment, since at 15 weeks of age, sucrose and stevia increased the percentage but it decreased with sucralose (HSD Tukey *p* = 0.001). When the groups of the 9th and 15th weeks of age were compared with the baseline group (3rd week of life), the cells producing IL-4 were found to be decreased at the end of the 12 weeks of consumption (*F* = 610, *p* = 0.001), as seen in Table 4.

In the case of IL-5, it increased at week 9 in the subgroups of Sucrose, Sucralose, and Stevia (HSD Tukey, *p* = 0.001). The subgroup of Sucralose kept a high IL-5, but it was significantly reduced in the Stevia subgroup (HSD Tukey, *p* = 0.001). Sucralose decreases the percentage of IL-10 at the 9th and 15th weeks of age (HSD Tukey, *p* = 0.001). On the other hand, stevia did not present a homogeneous behavior, since its secretion decreased at 9 weeks of age, but it increased significantly at week 15 (HSD Tukey, *p* = 0.001), as seen in Table 4.

#### 3.5.2. Concentration of Intracellular Cytokines Determined in Lamina Propria Lymphocytes

*Proinflammatory Cytokines (Th1)*. The consumption of stevia at 9 and 15 weeks of age caused a decrease in the CD4^+^ T cells secreting IFN-*γ* (HSD Tukey, *p* = 0.001), compared to sucralose, although it increased its production in week 9, which decreased at the end of week 15 of age. On the other hand, the T cells secreting TNF-*α* were increased in the subgroups of Sucralose and Stevia at 9 and 15 weeks of age (Table 5).

*Anti-Inflammatory Cytokines (Th2)*. The consumption of sucralose at 9 weeks of age caused an increase in the T cells profile secreting IL-4, IL-5, and IL-10 (HSD Tukey, *p* = 0.001). At week 15, the increase in IL-4 continued, but the secretion of IL-5 and IL-10 decreased. On the contrary, in the Stevia subgroup, interleukins 4, 5, and 10 decreased at 9 and 15 weeks of age (Table 5).

## 4. Discussion

The introduction of nonnutritive sweeteners in multiple commercial products aims to consume them and maintain the sweet taste that pleases the population but with a reduction in caloric intake. This as a consequence of the increasing prevalence of overweight and obesity with the purpose of decreasing and/or maintaining body weight. However, until now the data reported in relation to its effect are controversial particularly of sucralose and stevia.

The sweeteners provide a nice sweet taste to the palate that may be pleasant or not. In the majority of cases, the liking for the sweet taste adds to the preference of consumption, which is directly related to the habituation of taste receptors from early ages and the taste acquired by them. In the study carried out by Wang et al. [29], it was reported that sucralose increased food intake by activation of two mechanisms of action, first, by direct stimulation of sweet taste receptors and, second, by indirect stimulation of taste-independent neuronal mechanisms. In this study, the newly weaned mice increased their consumption of water with sweetener in a gradual manner and proportional to the time they had the water available with sweetener (5 hours a day). They also showed preference for the consumption of sucralose. In a study with obese children, it is shown that age is a determining factor in the preference for sweet taste; children prefer high concentrations of sweet taste particularly sucralose and aspartame [30]; this situation corresponds to the findings of this study, since young mice had preference for water sweetened with sucralose since weaning (3 weeks of age).

### 4.1. The Consumption of Noncaloric Sweeteners Reduced Food Consumption but Increased Body Weight

The results found in this study show that the groups supplemented with sweeteners decreased food consumption but increased body weight. This is consistent with the results of Mattes and Popkin in 2009 [31]; they report that the use of nonnutritive sweeteners increases weight and body mass index (BMI) in healthy sedentary subjects. Similarly, Yang in 2010 [32] reported an increase in body weight and BMI in healthy subjects who consumed beverages with three different sweeteners for a long time compared to those who consumed liquid without sweetener. In contrast, in the study by Uebanso et al. [33], they describe that mice supplemented with sucralose for 8 weeks at doses of 1.5 and 15 mg/kg of weight had no significant body weight gain compared with mice that did not receive sucralose. The results of this study show that mice prefer the consumption of sucralose and stevia, since they ingested a greater concentration of water with these sweeteners than water without sweeteners. Evidence suggests that the intensity of sweetness increases the preference of consumption of sweet flavors and increases the appetite [34, 35]. In rats supplemented with stevia and saccharin, it was shown that these rodents preferred the consumption of stevia compared with saccharin or liquid without sweetener [36].

Many studies have focused on the study of sweetener consumption in obese and diabetic patients. In both cases, the objective is to be able to include sweet foods without raising the caloric intake in their usual diet [37]. In fact, there are few studies that evaluate the effect of the consumption of sweeteners in a healthy population, much less in a very young population (newly weaned mice). However, in this study, differences in blood glucose concentration are shown with the consumption of sucralose and stevia, since sucralose reduces glycaemia and stevia increases it. The differences are observed between the groups at 3, 9, and 15 weeks of age and by type of sweetener. In addition, the stevia subgroup increased the HOMA index, evidencing insulin resistance, demonstrated by increasing its plasma concentration at 9 and 15 weeks of age; consequently, glucose concentrations increased in this subgroup. In contrast, the subgroup of sucralose did not increase glycaemia, in the long term (15 weeks of age), or modified the insulin secretion, unlike the HOMA index which decreased in these rodents. These results are controversial, since there are studies where it is reported that stevia increases insulin sensitivity [38] and induces antihyperglycemic effects in diabetic rats [39]. For example, in patients with type 2 diabetes supplemented with steviol glycosides for 3 months, plasma glucose concentrations did not increase [40]. In another study in diabetic patients supplemented with sucralose for 4 weeks, they found that the glycaemia did not increase when compared with the control group [41]. Most of the studies are focused on diabetes models, whether human or animal; this study was carried out in a model of healthy CD1 mice, since the model in diabetic subjects can vary parameters derived from the same pathology. In a model where the subjects already have a pathology established, the physiological conditions are different; this situation can explain the contradictory results. Another factor that intervenes in the maintenance of the homeostatic mechanisms of regulation of glucose metabolism is incretin hormones. These hormones are peptides secreted by the enteroendocrine cells of the small intestine [42], are released in response to food intake, and have an important effect on the control of satiety and homeostasis of plasma glucose. In this work, Glucose Insulinotropic Peptide (GIP) was quantified for its metabolic function [43, 44]. The results showed that sucralose reduces the secretion of GIP, glycaemia, and the HOMA index, but did not modify the insulin concentration; it also caused an increase in body weight with a reduction in feed intake, probably due to an increase in leptin secretion. There were no differences in the behavior of the GIP according to the route of administration used. For example, in humans, oral and intragastric administration of sucralose were performed and had no effect on the secretion of insulin, GLP-1, and GIP, as well as on gastric emptying compared with the administration of sucrose infusions, which increased the secretion of GLP-1 and GIP [45, 46]. In the same way, the administration of sucralose intraduodenally or by intragastric infusion did not produce changes in the rate of absorption of glucose in the small intestine or increase the glycemic response and incretin hormones levels in healthy humans [47]. Some studies have reported that oral supplementation with sucralose increases plasma levels of GIP and insulin in patients with obesity [48, 49], which suggests that the chronic use of nonnutritive sweeteners can develop a state of insulin resistance. Stevia, on the other hand, increased the secretion of GIP, insulin, the HOMA index, and leptin, which caused an increase in the body weight of the mice and blood glucose, although it maintained normal feed consumption. The elevation of GIP may be responsible for weight gain and glycaemia, since it participates in the genesis of obesity. Another probable explanation would be that the* Rebaudioside A* derived from the stevia that was used in this study is metabolized by the gut microbiota to steviosides and then transformed into glucose and a molecule of steviol; the final metabolite of stevia is the glucose that is absorbed in the intestinal epithelium [50], a situation that could generate the increase of glycaemia and therefore the elevation of GIP, insulin, and the HOMA index generating insulin resistance and causing an effect similar to sucrose. In addition,* Rebaudioside A* stimulates the release of insulin by inhibiting ATP sensitive K channels [17] favoring positive feedback of insulin increase but in turn increases resistance to it and therefore the glucose concentration rises. With this, we can conclude that depending on the type of active component of stevia that will predominate will be its metabolic effect.

### 4.2. Nonnutritive Sweeteners Modulate the Humoral Immune Response and Increase the Production of Proinflammatory Cytokines in the Small Intestine

The immune system is one of the cellular systems of the body that consumes the most energy, so that the nutritional status can influence both the innate and adaptive immunity by altering the number and functionality of the T and B lymphocytes in response to a nutritional imbalance [51]. Based on this, the study of the effect of nutrients in the various systems of the organism has given light to understanding how they affect or modify different metabolic and cell functions. In this work, the effect of sweeteners was evaluated first on their metabolic action and second on the immune system. The studies in relation to the effect of sweeteners have different results not yet so clear. With regard to stevia, it has been described that stevioside has beneficial effects on health, is considered an antihyperglycaemic, antihypertensive, and antioxidant agent, and has antitumor and anti-inflammatory capacity. Due to the fact that the human body is unable to digest it, it continues to be intact through the gastrointestinal tract to the small intestine, where it interacts with the intestinal microbiota and transforms into glucose and steviol [52, 53]. On the other hand, sucralose is not hydrolyzed, has a high solubility, and does not accumulate in the organism of experimental animals and humans. It is absorbed approximately 15% in humans and around 18% in mice; the rest is eliminated in the feces [54]. These low levels of absorption and the little evidence on the effects of sucralose* in vitro* have not demonstrated the influence of this sweetener on immune function in animal models, even at doses of 3 g/kg of body weight [55], nor is there evidence of adverse effects in studies of genotoxicity or carcinogenicity in the medium and long term [56]. It was observed that T and B cells and IgA^+^ were increased in Peyer's patches with the consumption of stevia, which demonstrates its immunomodulatory effect, improving the cellular response, with a marked increase in the anti-inflammatory interleukins of the type of IL-4 and IL-10. In lamina propria, the percentage of T cells was not modified, B cells and IgA^+^ plasma cells were increased, and paradoxically the percentage of IL-4, IL-5, and IL-10 cytokine-producing CD3^+^/CD4^+^ T cells was decreased.

Regarding the prolonged consumption of sucralose, the percentage of IgA^+^ decreased but increased the secretion of IL-5, B, and T cells which may suggest that sucralose inhibits the expression of IgA^+^ in plasma cells and increases the secretion of IL-5 in response to IgA^+^ deficiency. However, the behavior in the lamina propria at 15 weeks of age decreases the percentage of T cells and increases B cells and IgA^+^, with an increase in IL-4 but a decrease in IL-5 and IL-10. Because IL-4 promotes the differentiation of B lymphocytes and the production of antibodies [55, 56], this explains the increase in the percentage of B cells and IgA. In both compartments, sucralose and stevia decreased the secretion of INF-*γ* but significantly increased TNF-*α*. IL-10 inhibits the synthesis of IFN-*γ*; therefore its elevation could explain the reduction in the concentration of IFN-*γ* found. The data reported by Sehar et al. indicate that supplementation with 12.5 mg/kg of body weight of stevioside orally in normal mice increased the synthesis of antibodies up to 15.38% and increased the proliferation of B lymphocytes and T in culture with lipopolysaccharide stimulation (LPS) [57]; this agrees with the results reported in this study which favors the stance that stevioside has immunomodulatory properties. Exposure to high doses of water with sucralose (10 to 16 g/kg body weight) has no carcinogenic effects in mice; however, the intermediate product of the hydrolysis of sucralose 1-6-dichloro-1-6-dideoxy-D-fructose (1-6-DCF) has mutagenic capacity [58].

In another study in mice that were given steviosides intraperitoneally, it was reported that steviosides have anti-inflammatory and antiapoptotic effect, which concluded that the decrease in TNF-*α*, IL-1*β*, and IL-6 is dependent on the administered dose of steviosides; the higher the dose, the greater the inhibition of phosphorylation of NF-*κ*B and MAPK [59, 60]. In Balb/c mice, it was observed that the administration of steviosides with a nasal stimulus of lipopolysaccharide decreased lung damage and inhibited the secretion of proinflammatory cytokines and the phosphorylation of I*κ*B*α* and NF-*κ*B [61]. Steviosides can inhibit the release of TNF-*α* and IL-1*β* in THP-1 monocytes cells stimulated with LPS; however this effect is not observed with steviol. Stevioside is recognized by Toll-like Receptor 4 receptors (TLR4) by medium of the three glucose molecules in its structure and inhibits the release of proinflammatory cytokines [60, 62]. On the other hand, steviol is not recognized by the TLR4 and the recognition mechanism is through the membrane receptors for TNF-*α* and LPS, where it acts on the signaling pathway of the Nuclear Factor *κ*B (NF-*κ*B) inhibiting the phosphorylation of the I*κ*B*α* protein, which intervenes in the transcription of proinflammatory cytokines [59]. De Souza-Rocha et al. reported that supplementation with 4 mg/mL of sucralose in Wistar rats does not induce alterations in the morphology of red blood cells, which suggests that the consumption of this sweetener has no adverse effects on the cellular components of the organism [63]. Studies are still needed regarding the use of nonnutritive sweeteners that clarify other mechanisms of action in the organism.

## 5. Conclusions

The consumption of sucralose at an early age increases the preference for consumption of sweetened drinks at later ages and increases weight gain and also reduces the secretion of GIP and the HOMA index, with an increase in body weight. Stevia, on the other hand, increases glycaemia, GIP secretion, insulin, the HOMA index, and leptin causing body weight gain. Stevia consumption stimulates humoral immunity in Peyer's patches by increasing the percentage of B cells and IgA, with an increase in anti-inflammatory cytokines IL-4 and IL-10, although in lamina propria it triggers an inflammatory response due to increased TNF-*α* secretion. Sucralose decreases humoral immunity in Peyer's patches and decreases the percentage of IgA plasma cells; however, sucralose increases the humoral response in the lamina, by increasing the B cells and IgA and IL-4 cells, and reduces the inflammatory response by decreasing the secretion of TNF-*α*.

## Figures and Tables

**Table 1:** Preference of water consumption with and without sweetener in CD1 mice for 12 weeks.

	Control Mean ± SD mL (*n* = 8)	Sucrose Mean ± SD mL (*n* = 8)	Sucralose Mean ± SD mL (*n* = 8)	Stevia Mean ± SD mL (*n* = 8)	*p* ^*∗*^ value
*Baseline (4 weeks old)*					
Water without sweetener	123.9 ± 5.45	121.35 ± 9.35	116.2 ± 11.33	141.6 ± 15.82	0.001
Water with sweetener	----	45 ± 6.63	70.2 ± 2.76	63.95 ± 8.28	0.001
*Middle (9 weeks old)*					
Water without sweetener	235 ± 23.51	218.5 ± 6.94	221 ± 0.535	230 ± 7.75	0.053
Water with sweetener	----	86.65 ± 14	153 ± 4.3	96.3 ± 5.9	0.001^**∗**^
*Final (15 weeks old)*					
Water without sweetener	227 ± 32.07	206 ± 7.4	218 ± 11.7	231 ± 6.4	0.042^**∗**^
Water with sweetener	----	134.65 ± 4.3	191.7 ± 4	195.5 ± 4.4	0.001^**∗**^

Values represent the mean ± SD in mL (milliliters) of water with and without sweetener. One-way ANOVA was performed to compare the differences between subgroups. The differences were considered significant with a value of *p* < 0.05^*∗*^.

**Table 2:** Body weight, feed intake, and blood glucose concentration of CD1 mice supplemented with sweeteners for 12 weeks.

	*Baseline (3rd week)*	*Middle (9th week)*				*Final (15th week)*			
		CL	SUC	SUCL	ST	CL	SUC	SUCL	ST
	Mean ± SD (*n* = 8)	Mean ± SD (*n* = 8)	Mean ± SD (*n* = 8)	Mean ± SD (*n* = 8)	Mean ± SD (*n* = 8)	Mean ± SD (*n* = 8)	Mean ± SD (*n* = 8)	Mean ±SD (*n* = 8)	Mean ±SD (*n* = 8)
Body weight (g)	9.27 ± 0.08	30.60 ± 2.82	31.85 ± 3.1	32.75 ± 2.70	32.78 ± 2.31	34.47 ± 2.45	36.58 ± 2.8	37.1 ± 2.28^**∗**^	37.37 ± 3^**∗**^
Glycaemia (mg/dL)	116.5 ± 17	139 ± 22.77	134 ± 24	127 ± 7.9	140 ± 16.63	135 ± 13.8	137 ± 16	130 ± 10.32	142 ± 16
Feed intake (g)	88.6 ± 1.38	157.1 ± 9.51	149.8 ± 1.7^**∗**^	154.35 ± 0.267	149.3 ± 5.4^**∗**^	140.55 ± 11	125.5 ± 5.8^**∗**^	134.5 ± 4.2^**∗**^	139.45 ± 10

Values represent mean ± SD for body weight and feed intake in grams and mg/dL for blood glucose concentration. One-way ANOVA was used to compare subgroups with each other. The differences were considered significant with a *p* value < 0.05^*∗*^. CL = Control, SUC = Sucrose, SUCL = Sucralose, and ST = Stevia.

**Table 3:** Hormone profile of CD1 mice supplemented with sweeteners for 12 weeks.

	*Baseline (3rd week)*	*Middle (9th week)*				*Final (15th week)*			
		CL	SUC	SUCL	ST	CL	SUC	SUCL	ST
	Mean ± SD (*n* = 8)	Median ± SD (*n* = 8)	Median ± SD (*n* = 8)	Median ± SD (*n* = 8)	Median ± SD (*n* = 8)	Median ± SD (*n* = 8)	Median ± SD (*n* = 8)	Median ± SD (*n* = 8)	Median ± SD (*n* = 8)
GIP	123.2	37.14	66.57	75.59	86.59	127	121	93	145
Insulin	545.9	260	196	602	1282^a^	938	1143	939	1360^a^
Leptin	275.5	245	181	187	456	380	459	446	421
HOMA Index	181.4	340	132	240	451^a^	456	390	396	489^a^

The values represent the median in pg/mL for each determination; a nonparametric Kruskal-Wallis test^a^ was made of independent samples to compare the differences between the subgroups: the differences were considered significant with a *p* value < 0.05^*∗*^. CL = Control, SUC = Sucrose, SUCL = Sucralose, and ST = Stevia.

**Table 4:** Cytokine CD4^+^ T cell response in Peyer's patches from CD1 mice that consumed sweeteners for 12 weeks.

	*Baseline*	*Middle (9th week)*				*Final (15th week)*			
		CL	SUC	SUCL	ST	CL	SUC	SUCL	ST
	Mean ± SD % (*n* = 8)	Mean ± SD % (*n* = 8)	Mean ± SD % (*n* = 8)	Mean ± SD % (*n* = 8)	Mean ± SD % (*n* = 8)	Mean ± SD % (*n* = 8)	Mean ± SD % (*n* = 8)	Mean ± SD % (*n* = 8)	Mean ± SD % (*n* = 8)
IFN-*γ*	3.58 ± 0.074	2.01 ± 0.074	4.62 ± 0.096^**∗**^	2.37 ± 0.096^**∗**^	2.58 ± 0.074^**∗**^	2.52 ± 0.074	1.19 ± 0.096^**∗**^	2.21 ± 0.096	2.13 ± 0.074
TNF-*α*	2.67 ± 0.080	2.84 ± 0.058	3.08 ± 0.069	3.03 ± 0.080	1.57 ± 0.096^**∗**^	1.51 ± 0.058	1.56 ± 0.069	1.97 ± 0.080	1.74 ± 0.096
IL-4	6.52 ± 0.085	4.02 ± 0.106	2.65 ± 0.138^**∗**^	7 ± 0.085	3.59 ± 0.106^**∗**^	3.41 ± 0.096	4.14 ± 0.096	2.1 ± 0.085	5.91 ± 0.106
IL-5	4.37 ± 0.085	2.59 ± 0.08	3.4 ± 0.069^**∗**^	2.74 ± 0.085	3.02 ± 0.24^**∗**^	2.31 ± 0.08	2.25 ± 0.069	2.62 ± 0.085	1.36 ± 0.240
IL-10	3.17 ± 0.08	2.97 ± 0.069	3 ± 0.058	2.55 ± 0.096^**∗**^	2.15 ± 0.08^**∗**^	2.33 ± 0.069	5.58 ± 0.058	2.11 ± 0.096	5.53 ± 0.08

The values represent the mean ± SD of percentage of intracellular cytokines (CD3^+^/CD4^+^ T cells) determined by flow cytometry from Peyer's patches of CD1 mice at 3 (baseline), 9 (middle), and 15 (final) weeks of age. One-way ANOVA^*∗*^ was performed to compare the differences between the subgroups. The differences were considered significant with a value of *p* < 0.05^*∗*^. CL: Control, SUC: Sucrose, SUCL: Sucralose, ST: Stevia, IFN-*γ*: interferon-gamma, TNF-*α*: tumor necrosis factor-alpha, IL-4: interleukin 4, IL-5: interleukin 5, and IL-10: interleukin 10.

**Table 5:** Cytokine CD4^+^ T cell response in the lamina propria lymphocytes from CD1 mice that consumed sweeteners for 12 weeks.

	*Baseline*	*Middle (9th week)*				*Final (15th week)*			
		CL	SUC	SUCL	ST	CL	SUC	SUCL	ST
	Mean ± SD % (*n* = 8)	Mean ± SD % (*n* = 8)	Mean ± SD % (*n* = 8)	Mean ± SD % (*n* = 8)	Mean ± SD % (*n* = 8)	Mean ± SD % (*n* = 8)	Mean ± SD % (*n* = 8)	Mean ± SD % (*n* = 8)	Mean ± SD % (*n* = 8)
IFN-*γ*	15.52 ± 0.106	13.71 ± 0.053	12.06 ± 0.106	17.63 ± 0.106^**∗**^	11.03 ± 0.053^**∗**^	9.15 ± 0.053	3.73 ± 0.106	5.92 ± 0.106	5.36 ± 0.053
TNF-*α*	5.99 ± 0.106	4.47 ± 0.106	5.96 ± 0.096	7.31 ± 0.08^**∗**^	8.77 ± 0.096^**∗**^	4.47 ± 0.106	5.73 ± 0.096	7.41 ± 0.08	8.81 ± 0.096
IL-4	8.84 ± 0.08	7.54 ± 0.106	9.89 ± 0.096	18.29 ± 0.058^**∗**^	4 ± 0.053	6.82 ± 0.106	6.52 ± 0.096	7.6 ± 0.058	2.2 ± 0.053
IL-5	11.69 ± 0.106	13.95 ± 0.08	11.36 ± 0.08	19.55 ± 0.085^**∗**^	10.45 ± 0.08^**∗**^	11.23 ± 0.09	3.26 ± 0.08	6.45 ± 0.09	4.66 ± 0.07
IL-10	10.89 ± 0.08	8.95 ± 0.133	8.96 ± 0.053	16.56 ± 0.056^**∗**^	2.39 ± 0.08^**∗**^	9.5 ± 0.133	7.3 ± 0.053	8.72 ± 0.096	2.2 ± 0.08

The values represent the mean ± SD of percentage of intracellular cytokines (CD3^+^/CD4^+^ T cells) determined by flow cytometry from lamina propria of CD1 mice at 3 (baseline), 9 (middle), and 15 (final) weeks of age. One-way ANOVA was performed to compare the differences between the subgroups. The differences were considered significant with a value of *p* < 0.05^*∗*^. CL: Control, SUC: Sucrose, SUCL: Sucralose, ST: Stevia, IFN-*γ*: interferon-gamma, TNF-*α*: tumor necrosis factor-alpha, IL-4: interleukin 4, IL-5: interleukin 5, and IL-10: interleukin 10.
